# Sleep and cognitive performance of African-Americans and European-Americans before and during circadian misalignment produced by an abrupt 9-h delay in the sleep/wake schedule

**DOI:** 10.1371/journal.pone.0186843

**Published:** 2017-10-26

**Authors:** Gemma M. Paech, Stephanie J. Crowley, Charmane I. Eastman

**Affiliations:** Biological Rhythms Research Laboratory, Department of Behavioral Sciences, Rush University Medical Center, Chicago, Illinois, United States of America; Universita degli Studi di Bologna, ITALY

## Abstract

We conducted two studies of circadian misalignment in non-Hispanic African and European-Americans. In the first, the sleep/wake (light/dark) schedule was advanced 9 h, similar to flying east, and in the second these schedules were delayed 9 h, similar to flying west or sleeping during the day after night work. We confirmed that the free-running circadian period is shorter in African-Americans compared to European-Americans, and found differences in the magnitude and direction of circadian rhythm phase shifts which were related to the circadian period. The sleep and cognitive performance data from the first study (published in this journal) documented the impairment in both ancestry groups due to this extreme circadian misalignment. African-Americans slept less and performed slightly worse during advanced/misaligned days than European-Americans. The current analysis is of sleep and cognitive performance from the second study. Participants were 23 African-Americans and 22 European-Americans (aged 18–44 years). Following four baseline days (8 h time in bed, based on habitual sleep), the sleep/wake schedule was delayed by 9 h for three days. Sleep was monitored using actigraphy. During the last two baseline/aligned days and the first two delayed/misaligned days, beginning 2 h after waking, cognitive performance was assessed every 3 h using the Automated Neuropsychological Assessment Metrics (ANAM) battery. Mixed model ANOVAs assessed the effects of ancestry (African-American or European-American) and condition (baseline/aligned or delayed/misaligned) on sleep and performance. There was decreased sleep and impaired cognitive performance in both ancestry groups during the two delayed/misaligned days relative to baseline/aligned days. Sleep and cognitive performance did not differ between African-Americans and European-Americans during either baseline/aligned or delayed/misaligned days. While our previous work showed that an advance in the sleep/wake schedule impaired the sleep of African-Americans more than European-Americans, delaying the sleep/wake schedule impaired the sleep and cognitive performance of African-Americans and European-Americans equally.

## Introduction

Differences in sleep duration have been shown to exist between Blacks/African-Americans and Whites/European-Americans [1, 2]. Studies utilizing subjective reports have demonstrated that African-Americans report getting less sleep compared to European-Americans [3–7]. Subjective reports of sleep duration, however, may overestimate sleep duration [8]. While the finding that African-Americans obtain less sleep than European-Americans has also been observed in studies using objective measures of sleep such as polysomnography (PSG) or actigraphy, these studies were mostly conducted in older populations [9–15]. It is widely known that as adults age, sleep duration decreases [16], however, it is unknown if ancestry affects this decrease. It may be possible that the differences in sleep duration observed in these previous studies were exacerbated by the age of participants. Further, while these studies were conducted in participants homes, which can be beneficial to study sleep in a naturalistic setting, additional factors such as bed partners, neighborhood noise, and light exposure could differ and may have contributed to these differences in sleep duration. Indeed, there are some studies which suggest that differences in sleep duration between African-Americans and European-Americans may be due to socio-economic status (SES), environmental and social factors [6, 7].

There has been only one study, to our knowledge, that examined sleep in both a controlled laboratory environment and at home, with this study reporting no differences in sleep duration between African-Americans and Whites [17]. In this study, participants were also younger healthy adults with no sleep disorders or history of shiftwork. Likewise, Rao et al., [18] studied younger participants who slept in a controlled laboratory environment and also observed no differences in sleep duration between African-Americans and Whites. The findings of these two studies [17, 18] conflict with previous studies which have indicated that African-Americans obtain less sleep than Whites [3–7, 9–15]. These differences may be due to the controlled sleeping environment and/or younger age of participants, but call into question whether there are differences in sleep duration among people with different evolutionary ancestries.

We published two studies of circadian misalignment in non-Hispanic African and European-Americans. In the first [19] the sleep schedule was made earlier (advanced) by 9 h and in the second [20] the sleep schedule was made later (delayed) by 9 h. In our analysis of the sleep data from the first study we observed that, in these healthy younger adults sleeping within a controlled laboratory environment at habitual sleep times, there was little difference in sleep duration between African-Americans and European-Americans (published in this journal). We did observe, however, that the large, abrupt advance of the sleep/wake schedule for 3 days had a greater effect on the sleep and cognitive performance of African-Americans compared to European-Americans. African-Americans obtained approximately 6 h sleep during the three advanced sleep episodes, monitored with actigraphy, whereas European-Americans obtained approximately 7 h sleep during the first two advanced sleep episodes which declined to approximately 6.5 h during the third advanced sleep episode. African-Americans obtained significantly less sleep than European-Americans during the first two advanced sleep episodes. Given that sleep duration, particularly when restricted to less than 7 h, can result in subsequent cognitive performance impairments [21–23], it was not surprising that the shorter sleep obtained by African-Americans following the advanced sleep/wake schedule also negatively impacted cognitive performance However, it is unknown if the differences we observed during an advance in the sleep/wake schedule would also be observed when the sleep/wake schedule is delayed.

In our second study of circadian misalignment in African-Americans and European-Americans, the sleep/wake (light/dark) schedule was shifted later (i.e., delayed) by 9-h, as though participants had flown west across 9 times zones (e.g., from Chicago to Japan) [20]. A similar delay of sleep occurs when shift workers change from sleeping at night to sleeping during the daytime after a night shift. Findings relating to the relationship between the circadian period (τ) and the phase shift have been reported [20]. The current analysis presents the sleep, cognitive performance, sleepiness and mood data from this study. We compared these variables during delayed/misaligned days to baseline/aligned days and we compared the results from African-Americans to European-Americans. Based on our previous findings that African-Americans have a shorter free-running circadian period and their circadian rhythms delayed less after the three days of the delayed sleep-wake schedule [20], we hypothesized that a delay in the sleep/wake schedule would degrade the sleep and cognitive performance of African-Americans more than European-Americans.

## Materials and methods

### Participants

Participants were recruited with on-line ads and flyers from September 2014 through June 2016. An initial questionnaire excluded a majority of these individuals as they did not meet inclusion criteria: having a BMI less than 35, being a non-smoker, not being Hispanic or Latino and both biological parents being either Black/African-American or White/European-American. Follow up phone calls and in person interviews excluded other participants who did not meet more stringent inclusion criteria such as a clean drug screen and no reported health or sleep issues. We did not keep track of how many people applied to be in the study. There were 53 people who were enrolled and who signed consent forms three to four days before the start of the 14-day laboratory study which occurred between September 2014 and June 2016. Of these, 47 started the 14-day study, 46 completed the study and 45 were included in the current analyses. One participant (male) self-identified as Black/African-American and indicated that both parents and all four grandparents were Black/African-American. But we did not use his data because his ancestry DNA results (see below for methods) indicated he was 30% Sub-Saharan African, 56% European and 14% Indigenous American, and thus could not be included in the African-American group or the European-American group. The 45 participants were between 18 and 44 years old. There were 18 people who participated in the first study [19] and 9 to 33 months later participated in the second study [20].

Prior to entry to the study, participants completed a Family/Ancestor Questionnaire (S1 Appendix). This questionnaire asks participants to mark all of the following categories that applied to them, their biological parents, and all four grandparents: White, Black or African-American, Asian, Hispanic or Latino, European, Middle Eastern, Far East Asian, Indian Subcontinent, North African, Afro-Caribbean, American Indian or Alaska native, Native Hawaiian or other Pacific Islander, Other, Don’t Know. Participants self-identified as being either African-American (n = 23) or white (n = 22) and none self-identified as being “Hispanic or Latino”.

DNA samples were collected from Buccal (cheek) swabs and were analyzed (Ancestry *by* DNA, DNA Diagnostics Center, Fairfield, OH) to confirm self-reported ancestry. Greater detail about the Family/Ancestor Questionnaire and DNA samples is reported in Eastman et al. [20]. This company performed biogeographical ancestry estimates based on 176 ancestry informative markers, also known as population-specific alleles, which show large frequency differences between populations [24, 25]. Results were returned several weeks later with percentages for each subject in four categories: European, Sub-Saharan African, East Asian and Indigenous American (See the first table in [20]).

Participant demographics are shown in Table 1. Participants completed the Morningness-Eveningness Questionnaire (MEQ) [26] and the Munich Chronotype Questionnaire (MCTQ) [27]. As shown in Table 1, African-Americans were more morning-type (higher scores on the MEQ) than European-Americans (p<0.05). As shown in the first table of [20], there were 12 morning types and one evening type in the African-American group and five morning types and two evening types in the European-American group.

**Table 1:** Participant demographics.

	Combined	African-American	European-American
N	45	23	22
Sex (n)	23 F, 22 M	57% F	45% F
Age (years)	30.5 ± 7.0	32.1 ± 7.1	28.9 ± 6.5
BMI (kg/m^2^)	24.2 ± 3.7	25.0 ± 3.9	23.5 ± 3.4
SES	5.4 ± 1.6	5.4 ± 1.5	5.4 ± 1.7
MEQ	53.9 ± 8.9	56.5 ± 8.5	51.3 ± 8.8*
MSF	5.0 ± 1.2	4.8 ± 1.3	5.2 ± 1.1
Bedtime	00:09 ± 01:11	23:52 ± 01:08	00:27 ± 01:11
Wake-time	08:09 ± 01:11	07:52 ± 01:08	08:27 ± 01:11

Values shown as mean ± SD.

SES: Subjective Socioeconomic Score. MEQ: Morningness-Eveningness Questionnaire. MSF: Mid Sleep on Free days from Munich Chronotype Questionnaire score. Bedtime: scheduled baseline bedtime. Wake-time: scheduled baseline wake-time.

* Significant difference between African-Americans and European-Americans as determined by an independent t-test (p≤0.05).

Subjective socioeconomic status (SES) was determined using a socioeconomic status ladder [28] which consists of a 10-rung ladder. The top of the ladder represents those with the most money, most education and best jobs. The bottom represents those who have the least money, least education and worst or no job. Participants were instructed to place themselves on the ladder in terms of where they think they fell. This position was then translated into a score between 1 and 10, where 1 = bottom rung (i.e., lowest SES) and 10 = top rung (i.e., highest SES). In this way, higher scores are representative of a higher perceived SES.

All participants were physically and psychologically healthy as determined by a Health Information Questionnaire (S2 Appendix), a For Women Only Questionnaire (S3 Appendix), the Beck Depression Inventory [29] and the Minnesota Multiphasic Personality Inventory (MMPI-2) [30]. Participants also completed the Pittsburg Sleep Quality Index (PSQI) [31], the Epworth Sleepiness Scale (ESS) [32], the Berlin Questionnaire [33] and the Insomnia Severity Index [34] to determine if they had sleep disturbances.

Participants were non-smokers and were free from medication with the exception of six women (3 African-Americans and 3 European-Americans) who were taking oral contraceptives. Participants were low to moderate consumers of caffeine (≤ 300mg caffeine/day; equivalent to < 2cups coffee/day) and alcohol (≤ 3 standard drinks/day). Participants did not work night shifts in the month prior to the study. Participants were asked to abstain from caffeine in the four days preceding the study.

The study was approved from the Rush University Medical Center Institutional Review Board in accordance with standards set by the Declaration of Helsinki. All participants gave written informed consent to participate in the study. All were made aware that participation was completely voluntary and that they could withdraw at any time. Upon completion participants were given a financial compensation for their participation.

### Study design

**Fig 1:**
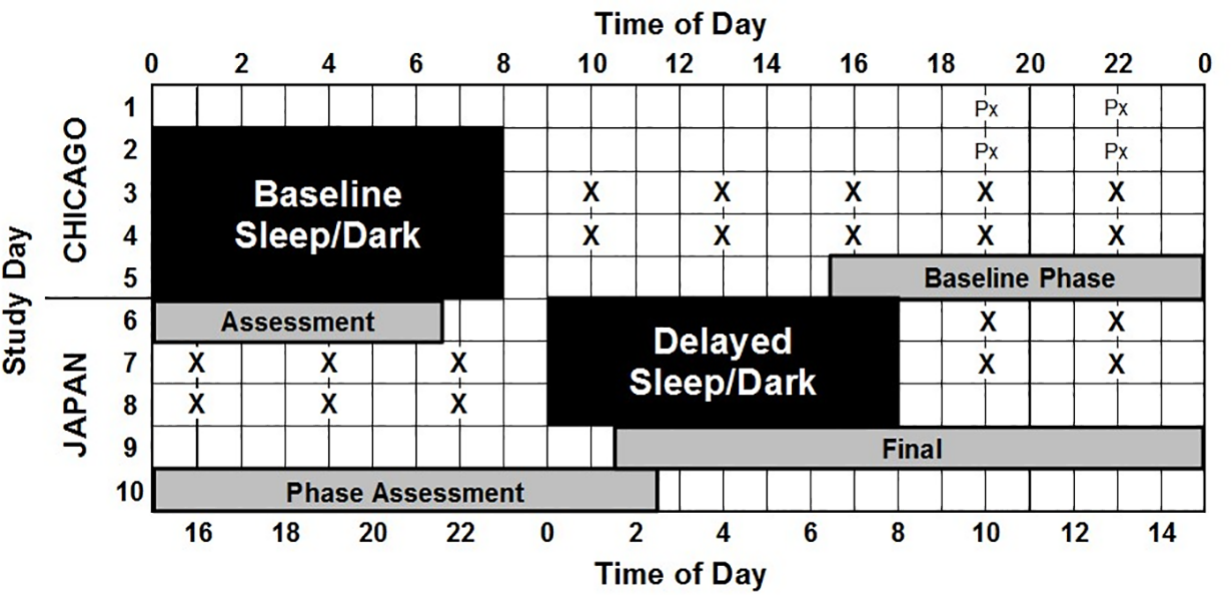
Protocol diagram. Time of day shown at the top is Chicago time and time of day shown on the bottom is Japan time (9-h earlier than Chicago time). Study day is shown on the left. Black shading shows timing of scheduled sleep periods. Schedules were individualized for each participant to best match their habitual sleep. This diagram shows the protocol for a participant on a 00:00–08:00 baseline sleep schedule. Days 1–5 were baseline during which participants remained on local, Chicago time (as indicated on the left). On days 7–9, the sleep schedule was shifted 9-h later (delayed), as though participants had traveled to Japan. The wall clocks in the bedrooms were changed to indicate the time in Japan. During baseline days the sleep schedule was aligned with each participant’s circadian rhythms, whereas during advanced days the sleep schedule was misaligned. “X” shows the timing of the Automated Neuropsychological Assessment Metrics (ANAM) performance battery and “Px” shows the timing of practice ANAM tests. Tests were given relative to each participants scheduled sleep times; 2 h after waking, and then every 3 h with a total of five tests per day. Light grey shading shows the timing of circadian phase assessments, during which the dim light melatonin onset (DLMO) was assessed.

The current analysis is of data from our study which involved a delay of the sleep/wake schedule [20], however, all methods are the same as our study which involved an advance in the sleep/wake schedule [19]. Both studies took place at the Biological Rhythms Research Laboratory in Chicago, USA. Participants completed the protocol in groups of two or three, usually with a mixture of African- and European-Americans in each group. The current report focuses on the last 10 days (labeled as days 1–10, Fig 1) of the larger 14-day study with the delay of sleep [20]. To see the protocol diagram for the full 14 days see Eastman et al. [20]. Participants remained in the laboratory for 14 days, with the exception of one 8-h break following the first baseline sleep (Fig 1, day 2) during which participants were free to leave the laboratory if they wished. While participants remained in the laboratory, they were continuously monitored by research staff. Caffeine and alcohol were not permitted while in the laboratory or during the 8-h break. Participants were reminded not to take any naps during the 8-h break. Upon re-entry to the laboratory participants were given a urine drug screen and were breathalyzed. While participants were not required to leave the laboratory, a majority of participants chose to do so.

Participants were scheduled to four baseline days with 8h time in bed (TIB) (Days 2–5 Fig 1). Baseline sleep schedules were tailored for each participant to best match their habitual schedule measured with sleep logs completed prior to the start of the study. Starting on day 6, the sleep-wake (light-dark) schedule was shifted 9 h later (i.e., delayed) as though participants had flown from Chicago to Japan which is 9 time zones west (Fig 1).

During baseline and delayed days, participants remained in a bedroom/testing suite. Participants had their own bedrooms, with separate, externally controlled lighting. Light levels were set to the maximum level (maximum ~ 500 lux, median; 66 lux at angle of gaze) during the first 10 h of wakefulness and dimmed to the lowest level (maximum < 100 lux, median; 17 lux) for the last 6 h of wakefulness. Rooms were completely dark during the 8-h sleep episodes. This simulates, as much as possible in our laboratory, the experience of people who may be exposed to brighter light for the first 10 h that they are awake, and then during the 6 h after sunset (when they are still awake) are only exposed to indoor artificial lighting which is less intense. Temperature was maintained at a consistent level (73 ± 2°F or 23 ± 1°C). Participants were allowed access to cell phones, electronic devices (e.g., laptops, tablets), and time pieces (e.g., watches) during waking episodes. All devices were turned off during performance testing and were removed from rooms during sleep episodes. Each bedroom had a wall clock indicating the ‘local’ time of either Chicago (baseline days) or Japan (delayed days).

Meals were served at regular intervals relative to each participant’s waking time starting on day 3; 1 h (breakfast), 6 h (lunch), and 12 h (dinner) after waking. Participants were allowed up to two small snacks (≤160 calories each) each day. Beginning 2 h after waking, participants completed a test battery every 3 h (Fig 1). Performance testing (described below) was conducted relative to each individual’s wake-time. There were five tests per day which were given during the last two baseline days and the first two advanced days (Fig 1). The schedule (meal timing, testing relative to waking time) remained the same during baseline and delayed days (Fig 1).

### Circadian phase

The method and results pertaining to circadian phase were reported in our previous publication [20].

**Fig 2:**
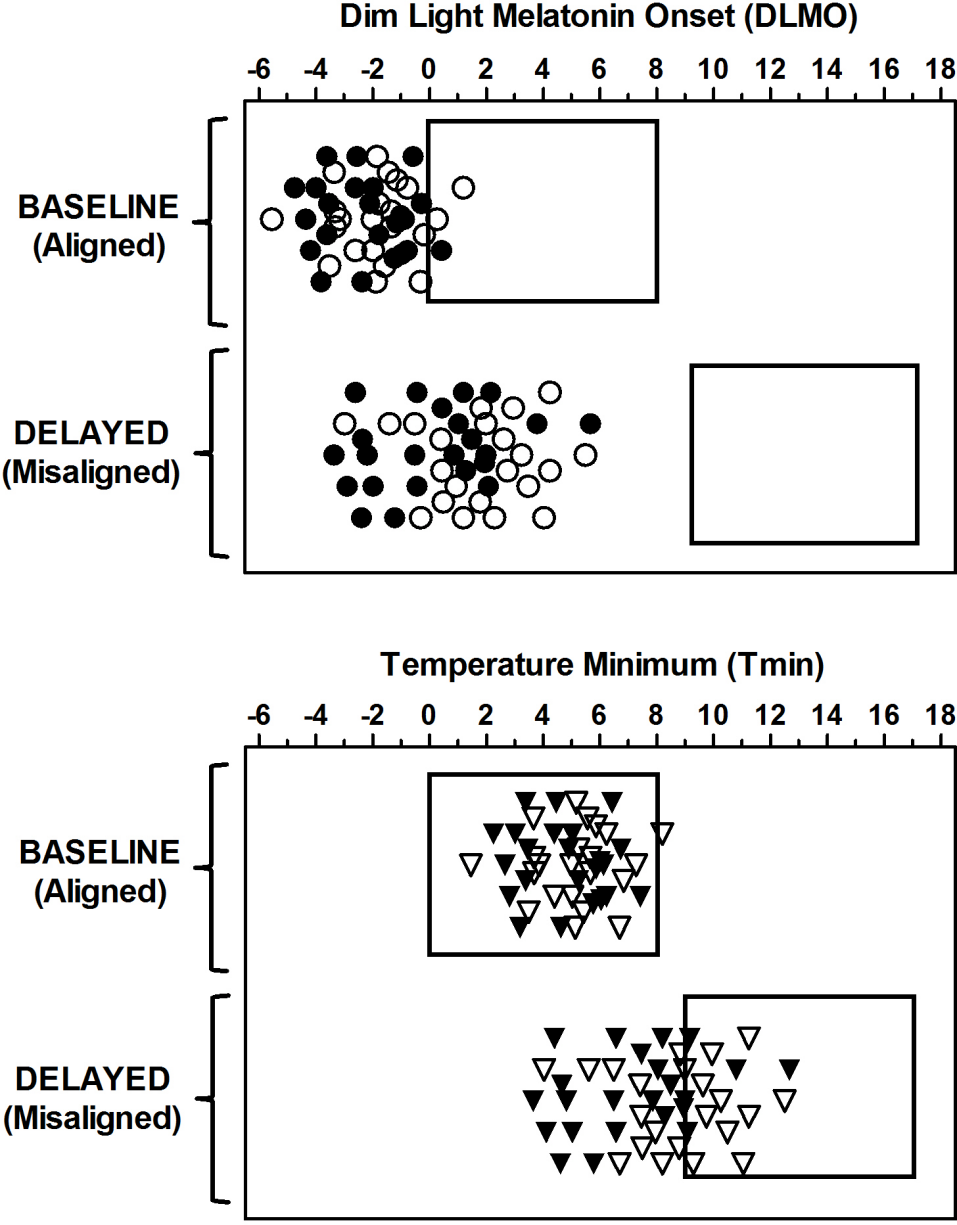
Dim light melatonin onset (DLMO) and estimated temperature minimum (Tmin) for each participant. DLMO was measured after the four baseline days on Chicago time (baseline, aligned) and after the three days on Japan time (delayed, misaligned). Rectangles show the timing of the sleep/dark periods. Top: Circles show the DLMO relative to the baseline bedtime, with 0 representing the timing of the start of the scheduled baseline sleep period. Bottom: Triangles show the Tmin relative to baseline bedtime. The Tmin was calculated as the DLMO + 7 h. Filled symbols represent African-Americans and open symbols are European-Americans. The DLMOs and Tmins were properly aligned to the sleep/dark periods during baseline (DLMOs before sleep and Tmins within sleep), but were misaligned relative to the sleep/dark period during delayed days. The vertical symbol placement is for visualization purposes and has no relationship to days.

Circadian phase markers are only presented here (Fig 2) to illustrate the enormous amount of circadian misalignment produced in this study. The method for determining circadian phase is described briefly below. On days 5 and 9 (Fig 1) participants remained seated in comfortable recliners under dim light conditions (<5 lux) during which the dim light melatonin onset (DLMO) was assessed. Saliva samples were collected every 30 mins using Salivettes (Starstedt, Newton, NC, USA). Samples were centrifuged, frozen, and later sent to Solid Phase Inc. (Portland Maine, USA) to be radioimmunoassayed for melatonin [35]. Based on prior work showing that the core body temperature minimum (Tmin) occurs approximately 7 h after the DLMO [36–38], Tmin was estimated by adding 7 h to the DLMO for illustration purposes (Fig 2).

### Sleep assessment

Sleep was measured via wrist actigraphy and sleep diaries. Participants wore activity monitors (Actiwatch Spectrum, Philips Respronics, Bend Oregon, USA) on their non-dominant wrist for the duration of the study. Sleep was recorded in 1-min epochs and data analyzed using the Philips Actiware-6 software package. Participants were required to complete their sleep diaries within 10 min of waking. Sleep diaries included information about sleep onset and offset times, and any wakefulness during the scheduled sleep episode. Early Morning Awakening (EMA) was the duration of time between the final awakening and scheduled wake time. In instances where a participant had been awake for longer than 2 h during the second half of the sleep episode, the EMA was manually calculated, even if they fell back asleep before the scheduled wake time. There were five occasions for four participants (3 African-Americans, 1 European-American) where the EMA was calculated manually. The primary outcome measure for both actigraphy and sleep diaries was total sleep time (TST). The use of actigraphy as a measure of TST has been validated against polysomnography (PSG) [39–41].

### Cognitive performance assessment

Participants completed the Automated Neuropsychological Assessment Metrics (ANAM) [42] test battery. The ANAM test battery, which was administrated on desktop computers, lasted approximately 20–30 min and consisted of nine different tasks. The ANAM tasks were completed in the following order: subjective sleepiness, mood, simple reaction time, code substitution learning, procedural reaction time, mathematical processing, matching to sample, code substitution delayed recognition, and Go/No-Go. All tasks within the ANAM battery, except the simple reaction time task which was a timed task, involved a set number of trials with the task ending upon completion of all trials. For tasks where a correct response was required (procedural RT, code substitution learning and delayed recognition, mathematical processing, and the matching to sample task) only correct responses were included in data analyses.

Subjective sleepiness was assessed using the Stanford Sleepiness Scale [43] which is a 7-point Likert scale where 1 = “feeling very alert, wide awake, and energetic” and 7 = “very sleepy and cannot stay awake much longer.” Participants selected the statement that best matched their current feelings of sleepiness. Participants rated their mood using an abbreviated 7-dimension mood scale [44], containing a set of 24 items. Using a scale of 0–6, where 0 = “not at all”, 3 (midpoint) = “somewhat”, and 6 = “very much”, participants rated each item based on their current state. Scores were grouped into seven mood dimensions: vigor (high energy level), happiness (positive disposition), depression (dysphoria), anger (negative disposition), fatigue (low energy level), anxiety (anxiety level), and restlessness (motor agitation).

Sustained attention and reaction time (RT) were measured using a 10-min simple RT task, akin to the psychomotor vigilance task (PVT) [45], with an interstimulus interval of 2–10 seconds. Participants were required to respond as quickly as possible (pressing the left mouse button) to a visual stimulus (asterisk) displayed in the center of a blank screen. Lapses were defined as RT > 500ms. Participants also completed a procedural RT task to assess processing speed and visuomotor RT when following a set of rules. In this basic block version of the task, a single digit between 2 and 5 was presented in the center of the screen. Participants were required to indicate whether the number presented was ‘low’ (2 or 3) with a left mouse click, or ‘high’ (4 or 5) with a right mouse click. Slow responses (SR)–comparable to lapses–were defined as responses exceeding the 90^th^ percentile of the cumulative distribution of each participant’s baseline responses [46]. Main outcome measures for the simple RT and the procedural RT tasks were the median RT and number of lapses or slow responses.

Two versions of the code substitution test were administered non-consecutively. The first version, code substitution learning, was similar to the Digit Symbol Substitution Test (DSST)[47]. In this test, a single digit-symbol pair was presented at the bottom of the screen. Participants were required to indicate whether the pair was correct (left mouse click) or incorrect (right mouse click) relative to a set of 9 defined digit-symbol pairs (i.e., the key) displayed at the top of the screen. Feedback was provided following each response. The second version, code substitution delayed recognition, was identical to the first, however the key was not displayed at the top of the screen. This test was presented several minutes after the learning version, after three intervening tests. Participants were required to determine whether the displayed digit-symbol pair was correct, based on the key presented earlier in the learning version. These tasks assessed sustained attention, visual scanning, associative learning and visual memory.

A mathematical processing task assessed basic computational skills and working memory. Participants were presented with a simple arithmetic problem (e.g., 4 + 8–5) and were required to indicate whether the answer was greater than (right mouse click) or less than (left mouse click) 5. Visuospatial working memory and processing was measured with a matching to sample task where a pattern 4 x 4 grid pattern with light and dark shaded cells was presented. Following a brief delay (5 sec) during which the screen was blank, two comparison grids were shown side-by-side. Participants indicated with a left or right mouse click, which grid matched the preceding grid. The primary outcome measure for the two code substitution tasks, mathematical processing, and matching to sample was the percent correct responses (number correct responses/number of trials*100).

Response inhibition was assessed with a Go/No-Go (GNG) task. One of two stimuli (“x” or “o”) were presented and participants were required to respond as quickly as possible to the “x” stimuli (i.e., “go”) but to do nothing, or inhibit the response (i.e., “no-go”), in response to the “o” stimuli. The number of correct responses (i.e., “hits”), incorrect responses (i.e., “false alarms”) and incorrect non-responses (i.e., “misses”) were extracted and a d-prime discriminability value was calculated (d’ = Z(hit rate)–Z(false alarm rate)). The d’ value, which was the primary outcome measure for this task, reflects the overall ability of the participant to discriminate between the go and no-go stimuli [48, 49].

Participants also completed a Columbia Jet Lag scale [50] each day prior to each sleep episode. This scale contains a set of nine items relating to sleepiness, fatigue, daytime alertness, and concentration. Participants rated how they had felt during the entire wake episode for each item using a scale of 0–4 where 0 = “not at all” and 4 = “extremely”. A total jet lag score was calculated from the sum of all the items.

### Data analysis

All data were analyzed using SPSS v.23 for Windows. Separate mixed model ANOVAs were performed to assess the main and interaction effects of condition (baseline or delayed) and ancestry (African-American or European-American) on sleep and performance. Regardless of main and interaction effects, separate mixed model ANOVAs were performed to assess the effects of ancestry on each day (sleep and Columbia Jet Lag Scale) or each hour after baseline wake time (cognitive performance measures). These additional analyses were performed to investigate how cognitive performance varied across hours of wakefulness or across days in the study. All models were performed on all variables (sleep and cognitive performance measures). As many of these variables were nearly identical to each other, only a subset of commonly used cognitive performance measures are reported in the results. Due to device error, actigraphy data was lost for two participants (1 African-American, 1 European-American). Data on the simple RT task for three individuals (all African-Americans) were excluded due to non-compliance. All models included participant ID as a random effect. Significance was assumed at p < 0.05.

## Results

### Circadian timing

Following baseline days, DLMOs and Tmins were in a normal phase relationship to sleep. With the exception of three participants, all DLMOs occurred before scheduled bedtime (Fig 2, top panel) and the estimated Tmins occurred within the sleep episode with the exception of one participant (Fig 2, bottom panel). In contrast, following the three delayed days on Japan time, DLMOs and Tmins for all participants were misaligned relative to the sleep episodes. While all the DLMOs occurred before the delayed sleep period, these occurred several hours earlier than usual (Fig 2, top panel). Further, most of the Tmins occurred before the scheduled sleep episode, i.e. at the wrong circadian phase (Fig 2, bottom panel). The magnitude of the phase delay for each individual is shown in Eastman et al. [20]. Most participants delayed between 0 h and approximately 4 h, with the largest delay being around 6 h. None of the participants delayed the whole 9 h, which would be required for complete circadian alignment with the delayed sleep/wake schedule. It should be noted that these phase delays were measured more than 24 hours after the last performance test. That is, the final phase assessment occurred after a full day with no performance measures. Therefore the phase delays that occurred during the time of performance testing and sleep measures would have been even less than that shown in Fig 2, or the fourth figure in [20].

### Sleep

**Fig 3:**
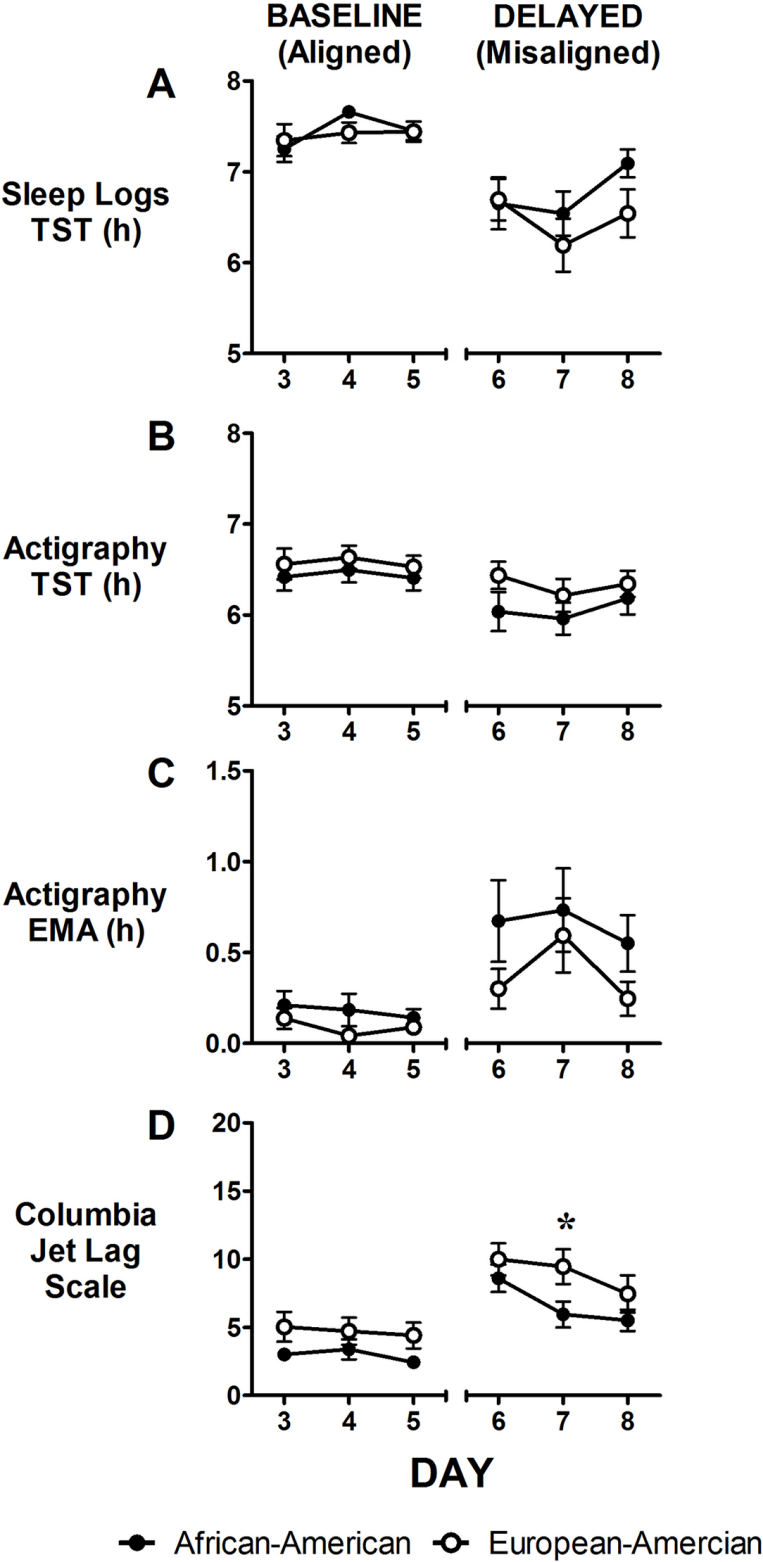
Total Sleep Time (TST), Early Morning Awakening (EMA), and Columbia Jet Lag Scale scores by study day. TST was measured using sleep logs (A) and actigraphy (B). EMA (C) was measured with actigraphy. Higher scores on the Columbia Jet Lag Scale (D) represent increased feelings of jet lag. Closed circles represent African-Americans and open circles represent European-Americans. Data are mean ± SEM. Baseline and delayed days (days 3–5 and days 6–8) were separated by a phase assessment period (refer to protocol, Fig 1). There were significant differences between baseline/aligned and delayed/misaligned days on all variables. There were no significant differences between African-Americans and European-Americans for any of the sleep measures, but there was one difference for Columbia Jet Lag Scale scores (p<0.05). N = 23 African-Americans and 22 European-Americans for sleep logs (A) and Columbia Jet Lag Scale (D) and N = 22 African-Americans and 21 European-Americans for actigraphy (B, C).

Fig 3 shows sleep duration for the last three baseline days (days 3–5) and the three delayed days (days 6–8). There were no main effects of ancestry or the interaction between ancestry and condition on any of the sleep variables; however, there were main effects of condition on all sleep variables (Tables 2 and 3). Compared to baseline/aligned days, TST was shorter, while EMA and Columbia Jet Lag Scale scores were higher during delayed/misaligned days (Tables 2 and 3). Across days, there were no differences between African-Americans and European-Americans except for Columbia Jet Lag Scale scores on day 7 (Fig 2) where European-Americans reported feeling more symptoms of jet lag.

**Table 2:** Main and interaction effects of ancestry and condition on sleep and cognitive performance.

	Ancestry			Condition			Ancestry*Condition		
Measures	DF	F	P	DF	F	P	DF	F	P
TST Logs	1,43	0.83	0.37	1,43	31.42	0.00*	1,43	0.67	0.42
TST Actigraphy	1,41	1.21	0.28	1,41	13.51	0.00*	1,41	0.61	0.44
EMA Actigraphy	1,41	2.63	0.11	1,41	15.79	0.00*	1,41	0.92	0.34
Columbia Jet Lag Scale	1,43	3.72	0.06	1,43	41.31	0.00*	1,43	0.15	0.70
SSS	1,43	2.03	0.16	1,43	45.75	0.00*	1,43	1.29	0.26
SRT Lapses	1,40	0.09	0.77	1,40	7.11	0.01*	1,40	0.27	0.61
SRT Median RT	1,40	0.03	0.85	1,40	5.74	0.02*	1,40	0.55	0.46
CSL % correct	1,43	0.83	0.37	1,43	7.07	0.01*	1,43	4.75	0.04*
CSD % correct	1,43	0.39	0.54	1,43	9.67	0.00*	1,43	0.01	0.91
GNG d-Prime	1,43	0.03	0.86	1,43	10.12	0.00*	1,43	1.92	0.17
MTH % correct	1,43	0.63	0.43	1,43	0.00	0.96	1,43	0.25	0.62
M2S % correct	1,43	0.05	0.83	1,43	1.00	0.32	1,43	0.01	0.93
ProRT SR	1,86	0.19	0.67	1,86	3.80	0.05	1,86	0.15	0.70
ProRT Medium RT	1,43	1.00	0.32	1,43	0.07	0.80	1,43	1.05	0.31
Mood—Vigor	1,43	1.74	0.19	1,43	35.5	0.00*	1,43	0.86	0.36
Mood- Happiness	1,43	6.00	0.02*	1,43	28.04	0.00*	1,43	0.06	0.81
Mood- Depression	1,43	1.73	0.20	1,43	0.50	0.48	1,43	1.81	0.19
Mood- Anger	1,43	1.45	0.24	1,43	3.31	0.08	1,43	0.22	0.64
Mood- Fatigue	1,43	3.10	0.09	1,43	34.74	0.00*	1,43	1.92	0.17
Mood- Anxiety	1,43	0.00	1.00	1,43	1.15	0.29	1,43	0.14	0.91
Mood- Restlessness	1,43	2.73	0.11	1,43	2.99	0.09	1,43	0.23	0.63

Ancestry was either African-American or European-American, and Condition was either Baseline (circadian rhythms aligned with sleep) or Delayed (circadian rhythms misaligned relative to sleep).

* Significant (p≤0.05) main effect of ancestry, condition, or their interaction.

TST: Total Sleep Time. EMA: Early Morning Awakening. SSS: Stanford Sleepiness Scale. SRT: Simple Reaction Time, lapses (RT <500ms.). CSL % correct: Code Substitution Learning percent correct responses. CSD % correct: Code Substitution Delayed percent correct responses. GNG d-Prime: Go/No-Go task d-prime score. MTH % correct: Mathematical Processing task percent correct responses. M2S % correct: Matching to Sample task percent correct responses. Pro RT SR: Procedural Reaction Time task number of Slow Responses (responses exceeding the 90^th^ percentile of each participant’s baseline responses). Pro RT Median RT: Procedural Reaction Time task median RT. SRT data from three participants was excluded and actigraphy data from two participants was lost (refer to methods).

**Table 3:** Means for sleep and cognitive performance measures for ancestry and condition.

	Ancestry		Condition	
Measure	African-American	European-American	Baseline	Shifted
TST Logs	7.11 ± 0.95	6.94 ± 1.08	7.43 ± 0.58	6.62 ± 1.18 ^b^
TST Actigraphy	6.25 ± 0.79	6.45 ± 0.69	6.51 ± 0.64	6.19 ± 0.82 ^b^
EMA Actigraphy	0.42 ± 0.75	0.24 ± 0.51	0.14 ± 0.27	0.52 ± 0.83 ^b^
Jet Lag Scale	4.82 ± 4.19	6.85 ± 5.77	3.81 ± 3.89	7.81 ± 5.42 ^b^
SSS	2.61 ± 1.64	2.94 ± 1.53	2.35 ± 1.21	3.19 ± 1.81 ^b^
SRT Lapses	8.30 ± 10.90	7.57 ± 10.90	6.94 ± 10.07	8.89 ± 11.60 ^b^
SRT Medium RT	325.86 ± 67.98	329.67 ± 62.28	322.87 ± 61.31	332.85 ± 68.26 ^b^
CSL % correct	94.72 ± 5.64	95.49 ± 3.29	95.44 ± 3.64	94.75 ± 5.47 ^b^^,^^c^
CSD % correct	80.24 ± 17.61	82.59 ± 18.26	82.86 ± 16.58	79.92 ± 19.16 ^b^
GNG d-Prime	2.73 ± 1.23	2.69 ± 1.14	2.80 ± 1.09	2.26 ± 1.28 ^b^
MTH % correct	94.37 ± 8.33	95.16 ± 6.34	94.74 ± 6.83	94.77 ± 8.00
M2S % correct	92.48 ± 9.15	92.84 ± 9.36	92.96 ± 8.22	92.36 ± 10.18
ProRT SR	3.33 ± 2.94	3.25 ± 2.70	3.10 ± 2.44	3.48 ± 3.15
ProRT Medium RT	498.96 ± 66.99	516.71 ± 81.16	508.01 ± 78.31	507.28 ± 71.08
Mood—Vigor	2.74 ± 1.43	2.29 ± 1.47	2.82 ± 1.40	2.21 ± 1.48
Mood- Happiness	3.95 ± 1.27	3.05 ± 1.53 ^a^	3.75 ± 1.40	3.27 ± 1.50
Mood- Depression	0.16 ± 0.42	0.40 ± 0.91	0.26 ± 0.70	0.29 ± 0.73
Mood- Anger	0.27 ± 0.69	0.50 ± 0.90	0.33 ± 0.74	0.45 ± 0.87
Mood- Fatigue	1.15 ± 1.31	1.59 ± 1.36	1.07 ± 1.12	1.66 ± 1.50
Mood- Anxiety	0.42 ± 0.70	0.42 ± 0.81	0.40 ± 0.73	0.44 ± 0.78
Mood- Restlessness	0.57 ± 0.88	0.95 ± 1.12	0.67 ± 0.90	0.84 ± 1.12

Values shown as mean ± SD.

^a^ Significantly different from African-American

^b^ Significantly different from baseline/aligned

^c^ Significant interaction between ancestry and condition

TST: Total Sleep Time. SSS: Stanford Sleepiness Scale. SRT Simple Reaction Time task, lapses (RT <500ms.).CSL % correct: Code Substitution Learning percent correct responses. CSD % correct: Code Substitution Delayed percent correct responses. GNG d-Prime: Go/No-Go task d-prime score. MTH % correct: Mathematical Processing task percent correct responses. M2S % correct: Matching to Sample task percent correct responses. Pro RT SR: Procedural Reaction Time task number of Slow Responses (responses exceeding the 90^th^ percentile of the cumulative distribution of each participant’s baseline responses). Pro RT Median RT: Procedural Reaction Time task median RT. Mood: seven mood sub-scales; vigor, happiness, depression, anger, fatigue, anxiety, and restlessness. Higher scores indicate worse performance for all measures except code substitution (learning and delayed) and Mathematical Processing, where higher scores indicate better performance.

### Cognitive performance

**Fig 4:**
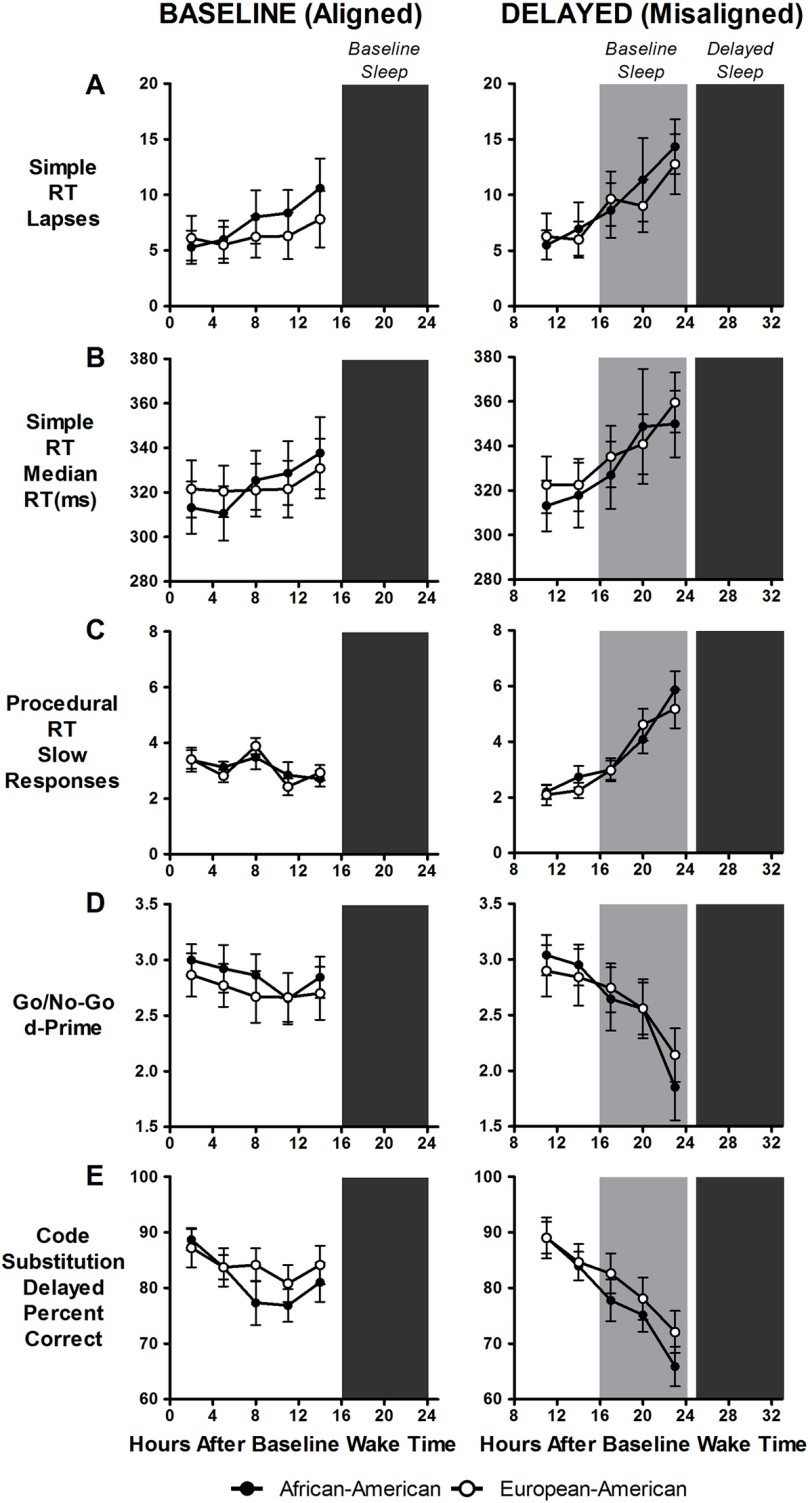
Cognitive performance during baseline/aligned and delayed/misaligned days. Performance was assessed on the simple reaction (RT) time task (A and B), procedural RT task (C), Go/No-Go task (D), and code substitution delayed task (E). Closed circles represent African-Americans and open circles represent European-Americans. Dark grey shading represents timing of scheduled sleep/dark episodes during both baseline/aligned and advanced (misaligned) days. Light grey shading on the right panels represents the previous baseline sleep episode. The last three cognitive performance tests (17, 20 and 23h after baseline wake time) during the delayed/misaligned days occurred when participants would normally be sleeping (i.e. during the scheduled baseline sleep/dark period). Lapses were defined as being RTs < 500ms, slow responses were responses exceeding the 90^th^ percentile of the cumulative distribution of each participant’s baseline responses, and d-Prime scores were the discriminability values indicating the overall ability to discriminate between the go and no-go stimuli. Data are mean ± SEM. Higher scores for A, B, and C represent worse cognitive performance. Lower scores for D and E represent worse cognitive performance. N = 20 African-Americans and 22 European-Americans (A and B). N = 23 African-Americans and 22 European-Americans (C, D and E). There were significant differences between baseline/aligned and delayed/misaligned days on all performance measures shown, but there were no significant differences between African-Americans and European-Americans on any of the cognitive performance measures.

**Fig 5:**
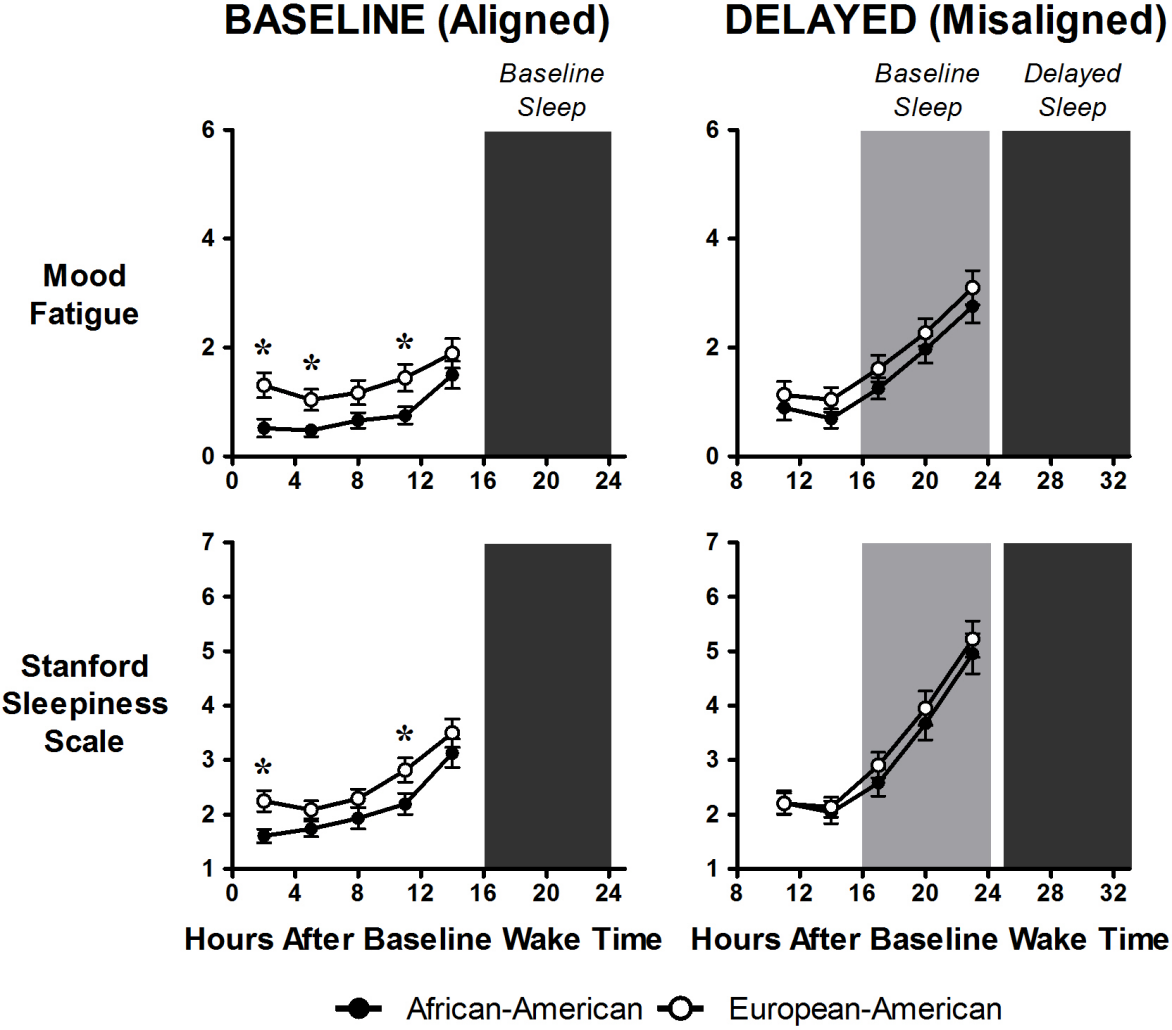
Subjective sleepiness and fatigue (low energy level) during baseline/aligned and advanced (misaligned) days. Closed circles represent African-Americans and open circles represent European-Americans. Dark grey shading represents timing of scheduled sleep/dark episodes during both baseline and advanced days. Light grey shading on right panels (advanced) represents the previous baseline sleep/dark episodes. Top panel shows subjective ratings on the fatigue mood dimension (low energy level) and the bottom panel shows subjective sleepiness (Stanford Sleepiness Scale). Data are mean ± SEM. Fatigue scores were on a scale of 0–6 and the Stanford Sleepiness Scale is a scale of 1–7. For both measures higher scores represent feeling more fatigue/sleepiness. N = 23 African-Americans and 22 European-Americans. * Significant difference (P≤0.05) between African-Americans and European-Americans as determined by mixed model ANOVAs. There were significant differences between baseline/aligned and delayed/misaligned days for both variables, and there were significant differences between African-Americans and European-Americans during baseline/aligned but not during delayed/misaligned days.

Fig 4 shows cognitive performance outcomes and Fig 5 shows subjective sleepiness and fatigue. There were no differences between African-Americans and European-Americans on any of the cognitive performance or subjective variables except for happiness (Table 2). On average, African-Americans reported feeling happier than European-Americans (Table 3). Compared to baseline/aligned days, cognitive performance and mood worsened on delayed/misaligned days on almost all measures (Tables 2 and 3).

During baseline/aligned days, cognitive performance remained relatively stable across hours of wakefulness, with slight decrements as the day progressed (Fig 4, left panels). Similarly, during baseline/aligned days, there was a slight increase in fatigue and sleepiness ratings as the day progressed (Fig 5, left panels). During delayed/misaligned days, however, cognitive performance worsened even more as the day progressed and was most impaired at times corresponding to the end of the baseline sleep episode (Fig 4, right panels). Likewise, during delayed/misaligned days, subjective sleepiness and fatigue also increased even more as the day progressed peaking around times corresponding to the end of the baseline sleep episode (Fig 5, right panel). There were no differences between African-Americans and European-Americans during either baseline/aligned or delayed/misaligned days on any of the performance measures (Fig 4). In contrast, during baseline/aligned days, European-Americans reported feeling slightly sleepier and more fatigued compared to African-Americans at several time points (Fig 5, left panels). There were no differences between the two groups in subjective sleepiness and fatigue during the delayed/misaligned days (Fig 5, right panels).

## Discussion

In the current study, the sleep/wake (light/dark) schedule was shifted 9-h later (i.e., delayed) as though participants had flown west across 9 time zones, similar to flying from Chicago to Japan. A similar large, abrupt delay in sleep occurs when shift workers have to sleep during the daytime after night shifts. During the baseline days of this study, when the sleep/wake schedule was aligned with the endogenous circadian rhythms, there were no differences in sleep or cognitive performance between African-Americans and European-Americans. Following the abrupt delay, when the sleep/wake schedule was misaligned with the endogenous circadian rhythms, sleep duration decreased and cognitive performance worsened compared to baseline/aligned days, however there were no differences between African-Americans and European-Americans. Thus, although an abrupt delay in the sleep/wake schedule resulted in decreased sleep and performance, these effects were not affected by ancestry. This study is, to our knowledge, the first that investigated whether there are differences between African-Americans and European-Americans in the sleep and performance following a delay in the sleep/wake schedule.

We hypothesized that European-Americans would obtain more sleep and perform better than African-Americans after the delay in the sleep/wake schedule, because their free-running circadian periods are longer and their circadian clocks delayed significantly more as shown by the DLMO (3.6 h compared to 2.4 h) [20]. Neither group, however, delayed close enough to the 9 h necessary for complete re-entrainment to the new sleep/wake (light/dark) schedule. This is also illustrated in Fig 2 of the current paper. Only a few of the estimated temperature minima reached the delayed sleep episode, which is considered enough circadian adaptation for improvements in sleep and performance [51]. Further, the delays seen in Fig 2 were measured more than 24 h after the last delayed sleep episode and last performance test, so the magnitude of the delays would be even less during the sleep episodes and performance tests. Therefore, both groups had similar degrees of circadian misalignment, and so it is not surprising that their sleep and performance were equally hindered.

Previous studies have reported differences in sleep duration between Blacks/African-Americans and Whites/European-Americans [9–15]. Conversely, in the current study, we did not observe any effects of ancestry on sleep duration. Current results are in line with those by Stepnowksy et al., [17] and Rao et al., [18], who also did not observe any differences in sleep duration between Blacks/African-Americans and Whites/European-Americans. Similarly, in our previous analysis of the sleep data from the study which involved a 9-h phase advance, there was little difference in TST between African-Americans and European-Americans during baseline/aligned days (published in this journal). The differences between groups with different ancestries observed in earlier studies [9–15] may be influenced by the sleeping environment (e.g., bed partners, light exposure, neighborhood noise) and/or the older age of participants. A controlled sleeping environment and younger participants could explain why we and others [17, 18], did not observe any ancestry differences in sleep duration. The role of aging and the sleeping environment on the sleep duration of African-Americans compared to European-Americans warrants further investigation.

Circadian misalignment has previously been shown to reduce sleep [52–54], and therefore the finding that on delayed/misaligned days TST was reduced compared to baseline/aligned days was expected. In contrast to our previous findings where we observed differences in TST during advanced/misaligned days between African-Americans and European-Americans (published in this journal), in the current study we did not observed any ancestry differences during delayed/misaligned days. While an advance in the sleep/wake schedule impaired the sleep of African-Americans more than European-Americans, delaying the sleep/wake schedule had similar effects on both groups.

A related observation is that mice subjected to a 6 h phase advance in the light/dark cycle once a week died much sooner than those subjected to a 6 h phase delay once a week [55]. This is surprising since mice have an average free-running period that is very short, in fact the average is less than 24 h (23.4 h and 23.6 h [56]), which should facilitate adjusting to a phase advance. Humans have an average free-running circadian period which is longer than 24 h, although it is shorter in African-Americans than European-Americans (24.07 h vs. 24.33 h [57]). A minority of people have circadian periods less than 24 h, and this is more common in African-Americans than European-Americans [57]. It appears that in mice and humans, an advance in the light/dark cycle is more detrimental than a delay, even when the circadian period is very short.

Although not significant, during delayed/misaligned days, African-Americans had descriptively longer EMA than European-Americans (Fig 3C). Both groups had significantly longer EMAs (spent more time awake around the end of the 8 h in bed) during the delayed/misaligned days than during the baseline/aligned days. It is expected that there will be early awakenings in those who fly west and in shift workers who sleep in the daytime after night shifts, because the end of scheduled sleep is so far from their temperature minima (see Fig 30–3 in [58] and see [51]). It makes sense that the African-Americans in this study would have even more trouble than the European-Americans sleeping as late as allowed, because their circadian rhythms did not delay as much. Although the difference in the magnitude of phase delays reached statistical significance, the difference in EMA did not.

In the current study, during the delayed/misaligned days, cognitive performance rapidly declined as time awake increased over the course of the day, with the worst cognitive performance around times corresponding to the end of the baseline sleep episode (Fig 4), highlighting the circadian and homeostatic influences on cognitive performance [54, 59–61]. There were no differences however, in cognitive performance between African-Americans and European-Americans on either baseline/aligned and delayed/misaligned days. We had previously shown some differences in cognitive performance between African-Americans and European-Americans following an advance in the sleep/wake schedule, and we suggested that these differences could be attributed to the TST differences we also observed between the groups (published in this journal). As we did not observe any differences in TST between African-Americans and European-Americans in the current study, it is not surprising that there were also no differences in cognitive performance following a delay in the sleep/wake schedule.

Subjective sleepiness and fatigue did not differ between African-Americans and European-Americans, except during baseline/aligned days when European-Americans reporting higher levels of sleepiness and fatigue (Fig 5). European-Americans also reported feeling less happy than African-Americans. It is difficult to determine the cause for these differences, as they–at least for subjective sleepiness and fatigue–occurred during baseline/aligned days, when differences between groups were not expected. It should be noted, however, that these were small differences and are not likely to be clinically significant [44].

Results from the current study suggest that a delay in the sleep/wake schedule invokes a similar decrease in sleep and cognitive performance in both African-Americans and European-Americans; however, there are some limitations that need to be considered. First, participants in the current study were young, healthy adults and the study was completed in a controlled laboratory environment. As such, results may not be fully applicable to the wider population and different sleeping environments. Second, we did not perform power analyses for the cognitive performance and sleep measures, and there was a large amount of variability in several of our measures, particularly cognitive performance measures, which may have reduced the overall statistical power of the study. Finally, another limitation is that in the current study sleep was assessed with sleep logs and actigraphy. Actigraphy, which although highly correlated with PSG, may not always be accurate in determining periods of still wakefulness from periods of sleep [39–41].

Despite these limitations, the current study has a key advantage over previous work. While previous studies relied on self-reports of race/ethnicity [3–7, 9–15], which can be very broad and is often not well defined [62], the current study used genetic testing in conjunction with self-identification of race/ancestry. Without this genetic testing, we would not have known that one participant who had self-identified as being African-American actually had more European than Sub-Saharan African ancestry. This finding highlights the importance of using an objective measure of ancestry (e.g., genetic testing) in addition to self-assessments to establish the ancestry of participants [63].

Current results indicate that delaying the sleep/wake schedule by 9 h has similar effects on the sleep and performance of both African-Americans and European-Americans. Delaying the sleep/wake schedule, similar to flying west over several time zones or sleeping during the day after night work, resulted in a misalignment between the endogenous circadian rhythms and the sleep/wake schedule, reduced TST and caused cognitive performance impairments. While the effects were similar between African-Americans and European-Americans, results may have greater implications for African-Americans who are more likely to work night shifts [64–66].

## Supporting information

S1 Appendix (PDF)

S2 Appendix (PDF)

S3 Appendix (PDF)

S1 Excel Data File (XLSX)
